# Supplementary material for: Bayesian network modeling of patterns of antibiotic cross-resistance by bacterial sample source
Source: Commun Med (Lond). 2023 May 2;3:61. doi: 10.1038/s43856-023-00289-7 (PMC10154291; doi:10.1038/s43856-023-00289-7)
Supplement: Supplementary file 1 — Description of Additional Supplementary Files [file 43856_2023_289_MOESM1_ESM.pdf]

Description of Additional Supplementary Files

File Name: Supplementary Data 1

Description: Summary statistics.

File Name: Supplementary Data 2

Description: *E coli* urine parameter estimates and 95% credible intervals.

File Name: Supplementary Data 3

Description: *E coli* wound parameter estimates and 95% credible intervals.

File Name: Supplementary Data 4

Description: *E coli* aerobic blood parameter estimates and 95% credible intervals.

File Name: Supplementary Data 5

Description: *K pneumoniae* urine parameter estimates and 95% credible intervals.

File Name: Supplementary Data 6

Description: *K pneumoniae* wound parameter estimates and 95% credible intervals.

File Name: Supplementary Data 7

Description: *K pneumoniae* aerobic blood parameter estimates and 95% credible intervals.

File Name: Supplementary Data 8

Description: *P aeruginosa* urine parameter estimates and 95% credible intervals.

File Name: Supplementary Data 9

Description: *P aeruginosa* wound parameter estimates and 95% credible intervals.

File Name: Supplementary Data 10

Description: *P aeruginosa* sputum parameter estimates and 95% credible intervals.

File Name: Supplementary Data 11

Description: *P mirabilis* urine parameter estimates and 95% credible intervals.

File Name: Supplementary Data 12

Description: *P mirabilis* wound parameter estimates and 95% credible intervals.

File Name: Supplementary Data 13

Description: *P mirabilis* aerobic blood parameter estimates and 95% credible intervals.

File Name: Supplementary Data 14

Description: *S aureus* wound parameter estimates and 95% credible intervals.

File Name: Supplementary Data 15

Description: *S aureus* aerobic blood parameter estimates and 95% credible intervals.

File Name: Supplementary Data 16

Description: Parameter estimates (ln(OR)) and their 95% credible intervals, for arcs between pairs of antibiotics, by bacteria and sample source, where both antibiotics appear in more than one sample source for a given bacterial species.
