# Supplementary material for: Bayesian network modeling of patterns of antibiotic cross-resistance by bacterial sample source
Source: Commun Med (Lond). 2023 May 2;3:61. doi: 10.1038/s43856-023-00289-7 (PMC10154291; doi:10.1038/s43856-023-00289-7)
Supplement: Supplementary file 2 — Supplementary Information [file 43856_2023_289_MOESM2_ESM.pdf]

Supplementary Information

Table S1: Antibiotic resistance tests excluded from analysis due to redundancies or missing data, along with their tetrachoric correlations with highly-correlated included variables

Dataset	Antibiotic removed	Antibiotics it is correlated with	Tetrachoric correlation range
E.coli	AMP	CAZ, CRO, CXM, LEX	0.80 - 0.93
E.coli	CRO	CAZ	1.00
E.coli	CXM	CAZ	0.99 - 1.00
E.coli	LEX	CAZ	0.98 - 0.99
K. pneumoniae	AMC	CAZ, CIP, CRO, CXM, GEN, LEX, TZP	0.70 - 0.99
K. pneumoniae	CIP	OFX	0.89 - 1.00
K. pneumoniae	CRO	CAZ	0.98 - 1.00
K. pneumoniae	CXM	CAZ	0.99 - 1.00
K. pneumoniae	LEX	CAZ	0.98 - 0.99
P. aeruginosa	CIP	GEN	0.88 - 0.98
P. aeruginosa	PIP	CAZ, TZP	0.90 - 0.99
P. aeruginosa	IPM	MEM	0.94 - 0.99
P. mirabilis	AMP	CRO, CXM, GEN	0.87 - 1.00
P. mirabilis	CAZ	CRO, CXM	0.97 - 1.00
P. mirabilis	CIP	OFX	0.97 - 1.00
P. mirabilis	LEX	CRO, CXM	0.97 - 1.00
S. aureus	CIP	OXA	0.86 - 0.96
S. aureus	ERY	CLI	0.98 - 0.99

Note: AMC, amoxicillin/clavulanate; AMP, ampicillin; CAZ, ceftazidime; CIP, ciprofloxacin; CLI, clindamycin; CRO, ceftriaxone; CXM, cefuroxime; ERY, erythromycin; Fusid, fusidic acid; GEN, gentamicin; IPM, imipenem; LEX, cefalexin; MEM, meropenem; MUP, mupirocin; OFX, ofloxacin; OXA, oxacillin; PIP, piperacillin; SXT, sulfamethoxazole/trimethoprim, TZP, piperacillin/tazobactam.

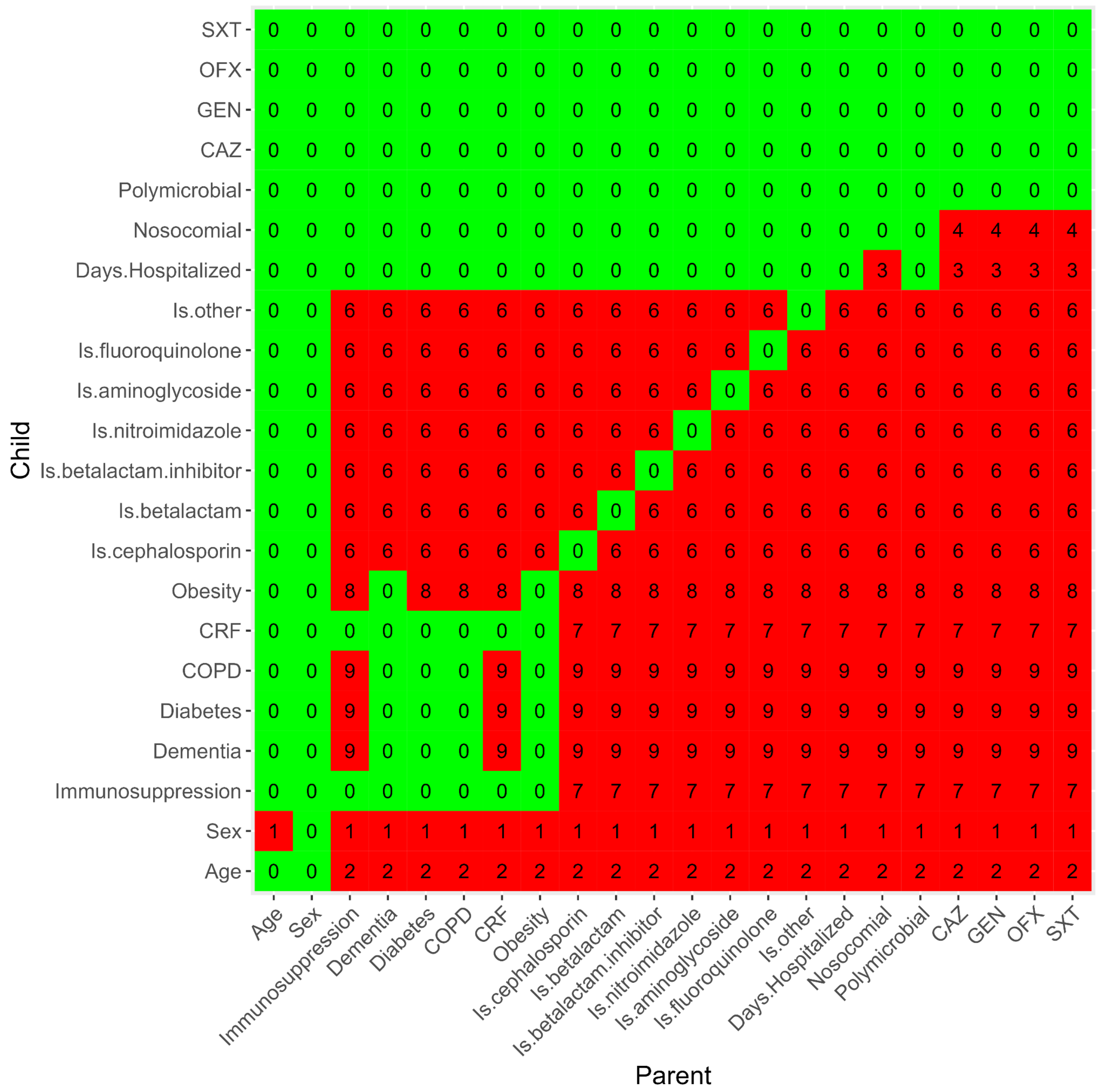

Figure S1: Sample matrix of banned arcs from parent node (columns) to child nodes (rows), denoted by the color red, with permitted arcs in green, from the *E coli* urine model. Numbers in red refer to the restrictions described in the text (Methods, statistical analysis).

Table S2: The maximum number of parents sufficient to reach the maximum likelihood, the number of arcs in this model, and the number of arcs present in at least 50% of the simulations, representing the final models presented, for each of the 5 bacteria analyzed.

Bacteria	Site	Max parents needed	Initial # of arcs	Arcs in 50% of simulations
E coli	Urine	7	42	39
	Wound	5	26	26
	Aerobic blood	6	19	17
K pneumoniae	Urine	5	22	22
	Wound	3	11	11
	Aerobic blood	3	9	9
P aeruginosa	Urine	6	19	18
	Wound	4	17	16
	Sputum	5	18	18
P mirabilis	Urine	4	17	17
	Wound	4	18	18
	Aerobic blood	2	7	4
S aureus	Wound	4	26	25
	Aerobic blood	2	8	7

Note: Initial number of arcs is for models where variables have been removed due not being connected to the resistance variables, or in the case of Ecoli urine, variables removed to allow models to run within a reasonable (2 weeks) amount of time.

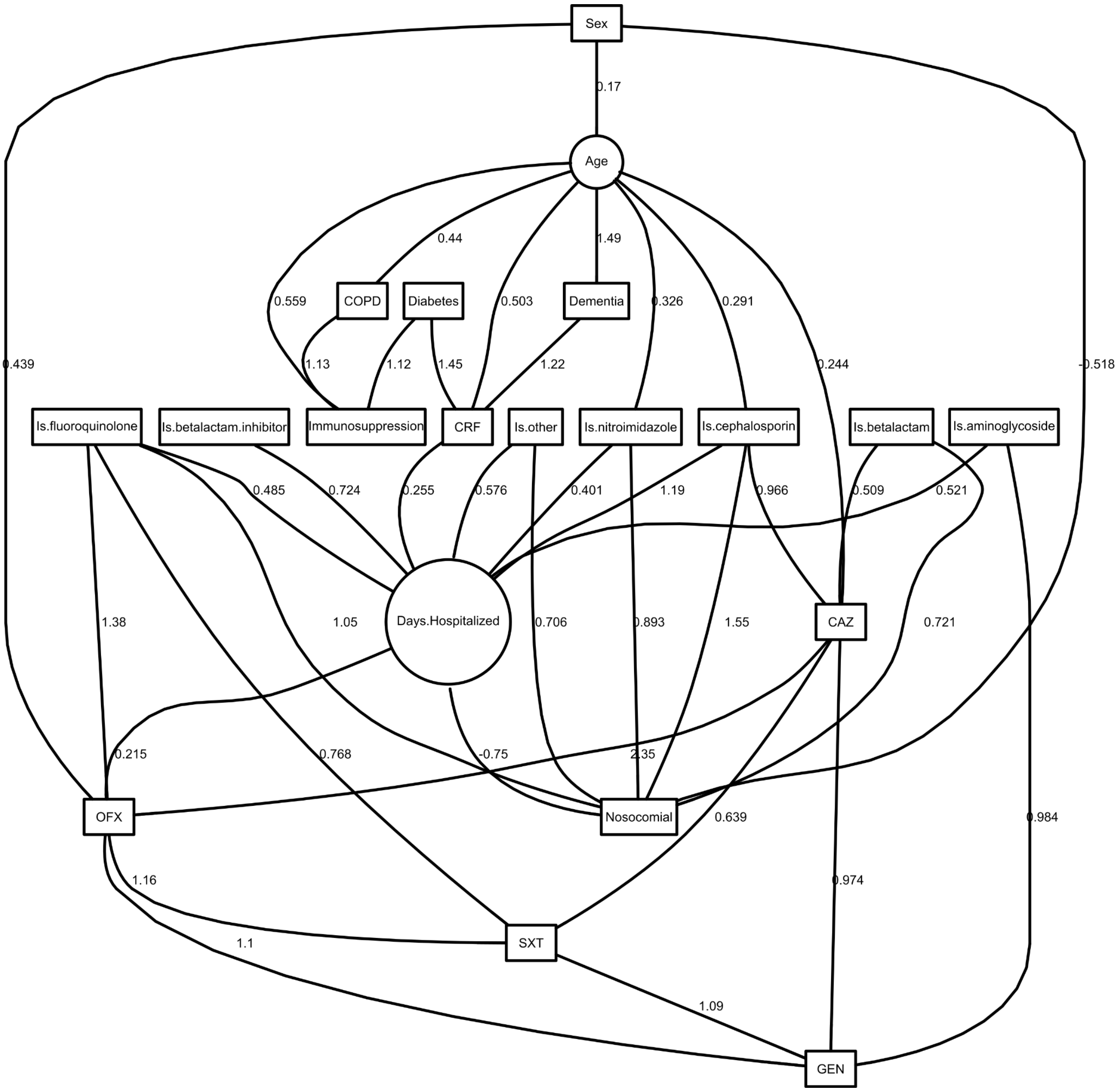

Figure S2: Final DAG for *E coli* in urine.

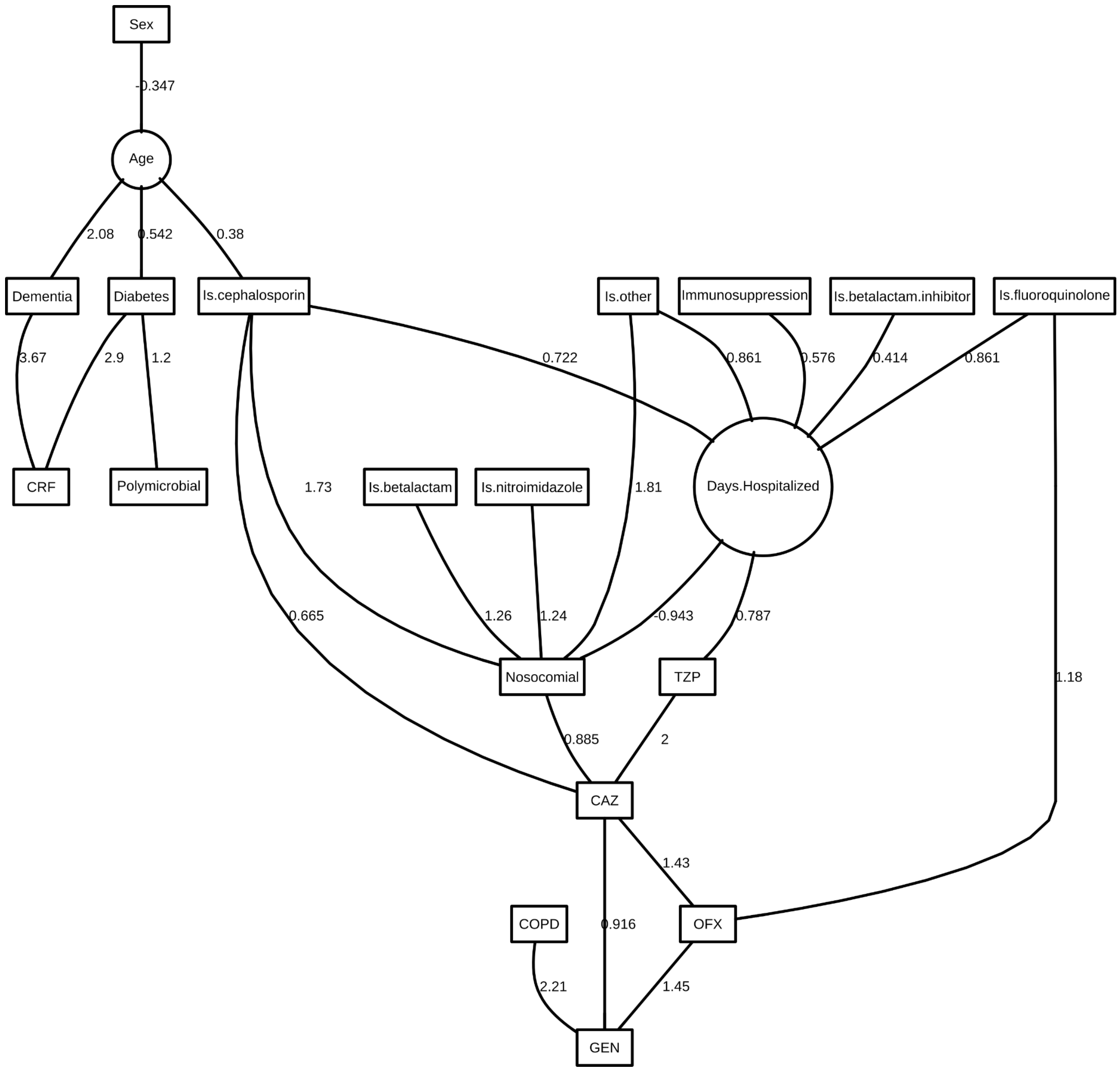

Figure S3: Final DAG for *E coli* in wound.

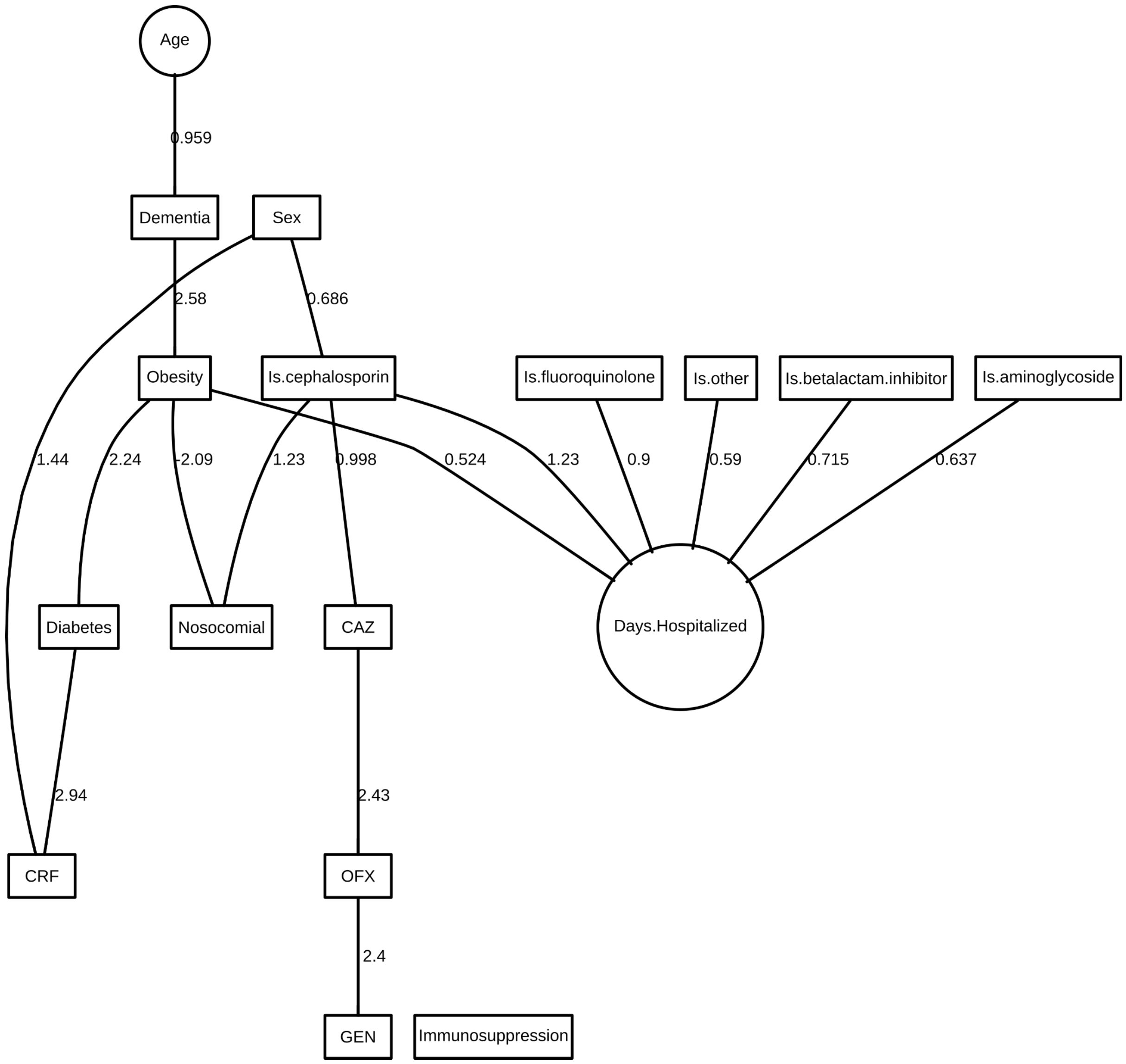

Figure S4: Final DAG for *E coli* in aerobic blood.

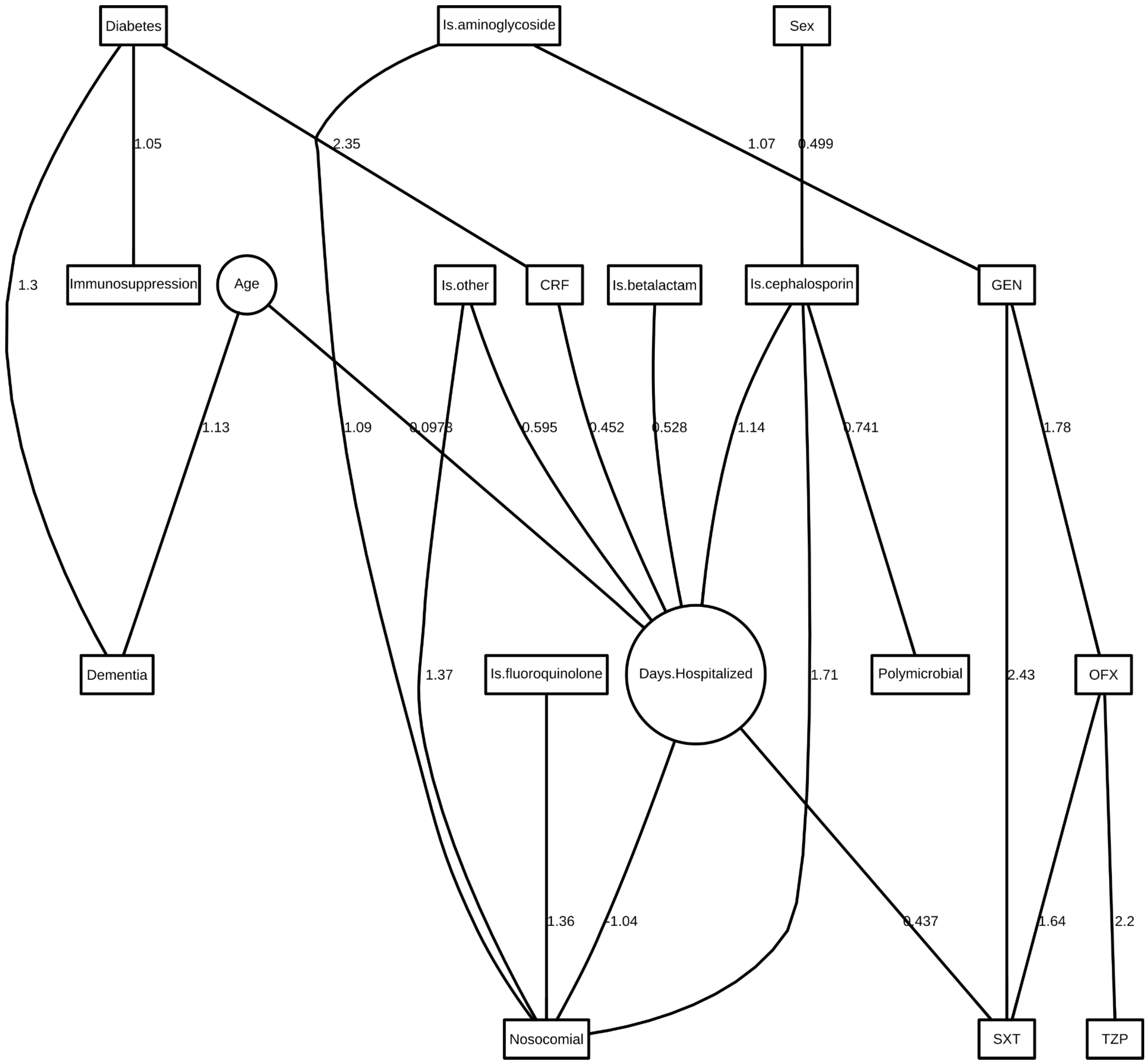

Figure S5: Final DAG for *K pneumoniae* in urine.

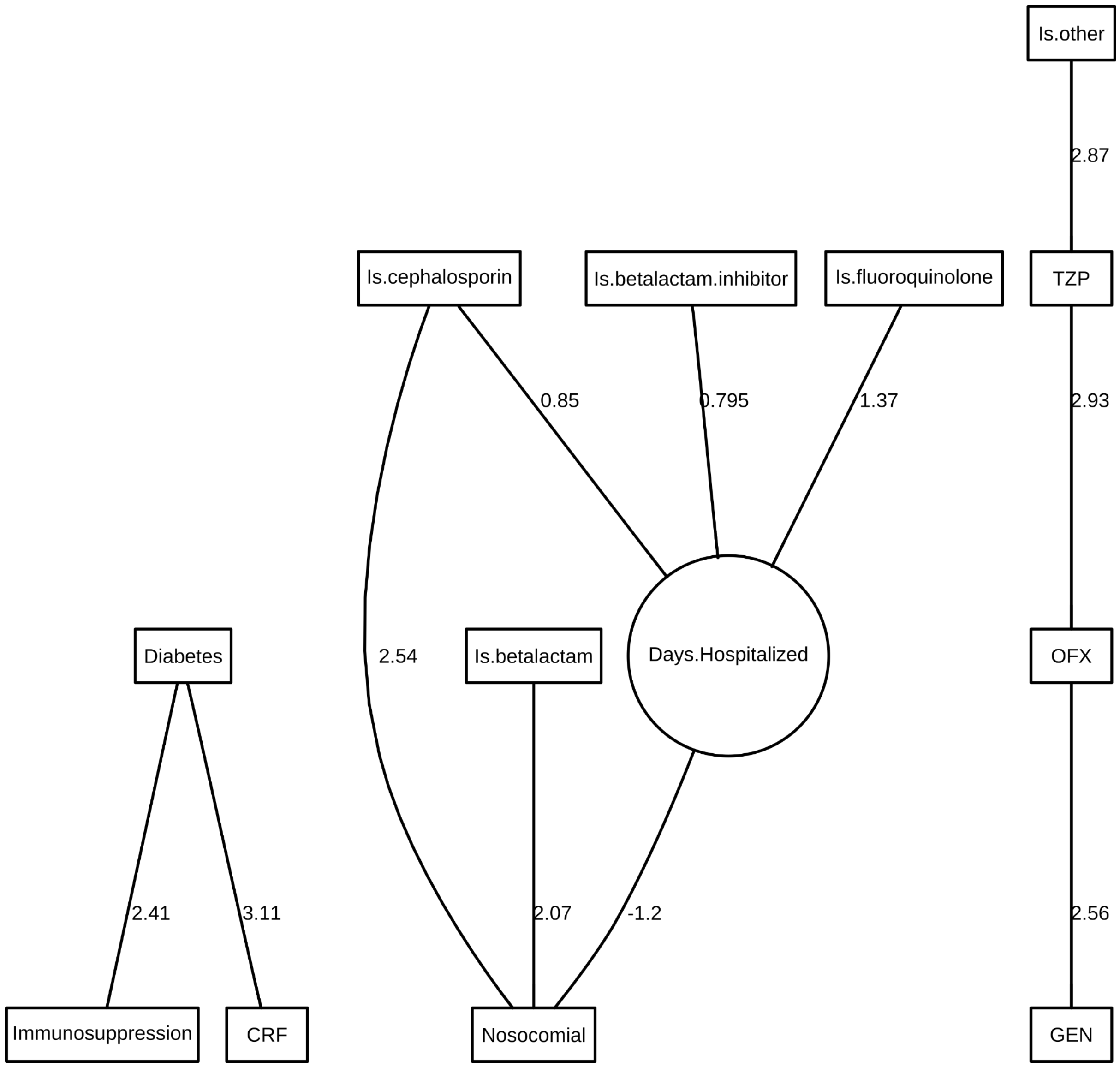

Figure S6: Final DAG for *K pneumoniae* in wound.

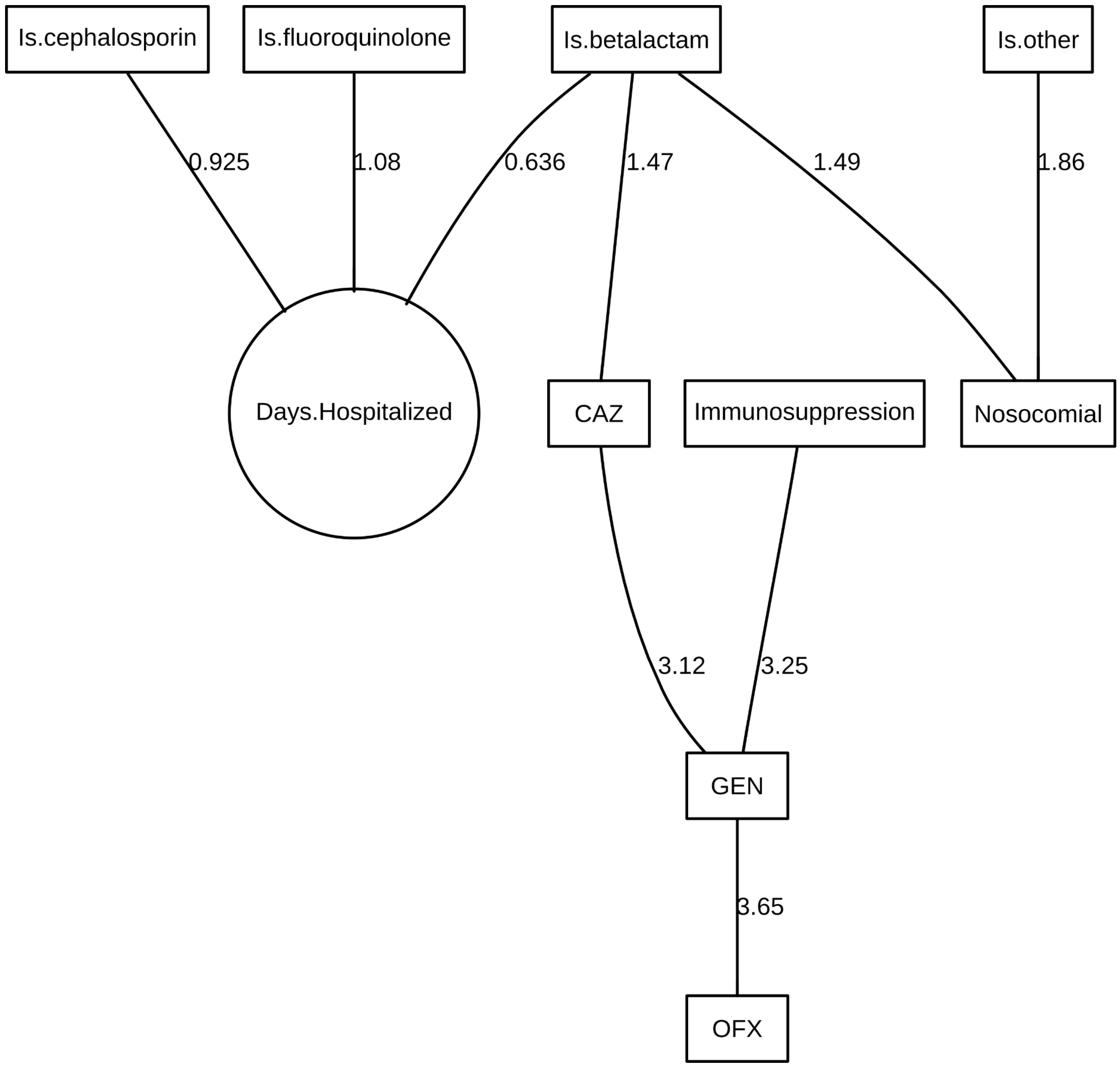

Figure S7: Final DAG for *K pneumoniae* in aerobic blood.

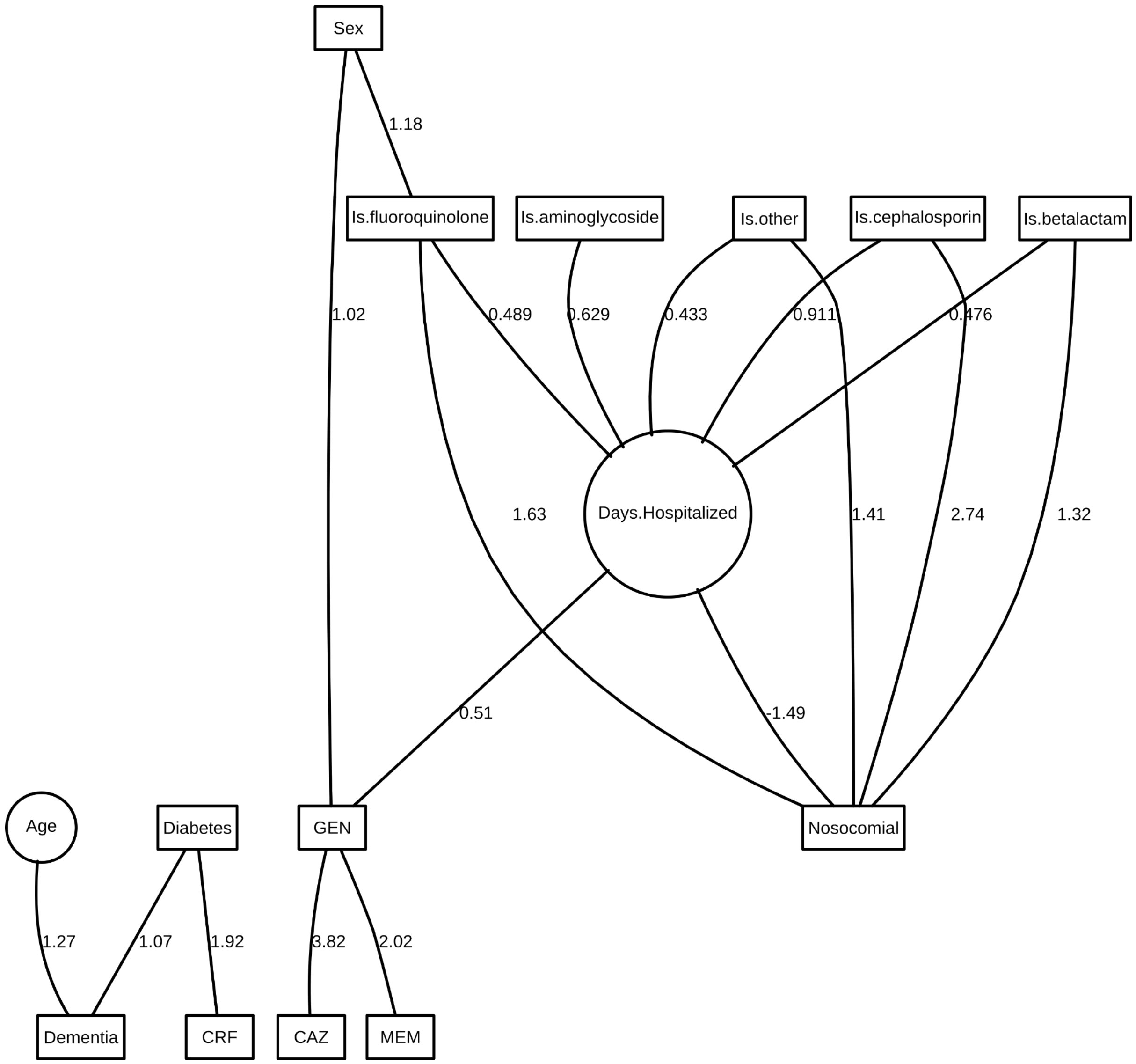

Figure S8: Final DAG for *P aeruginosa* in urine.

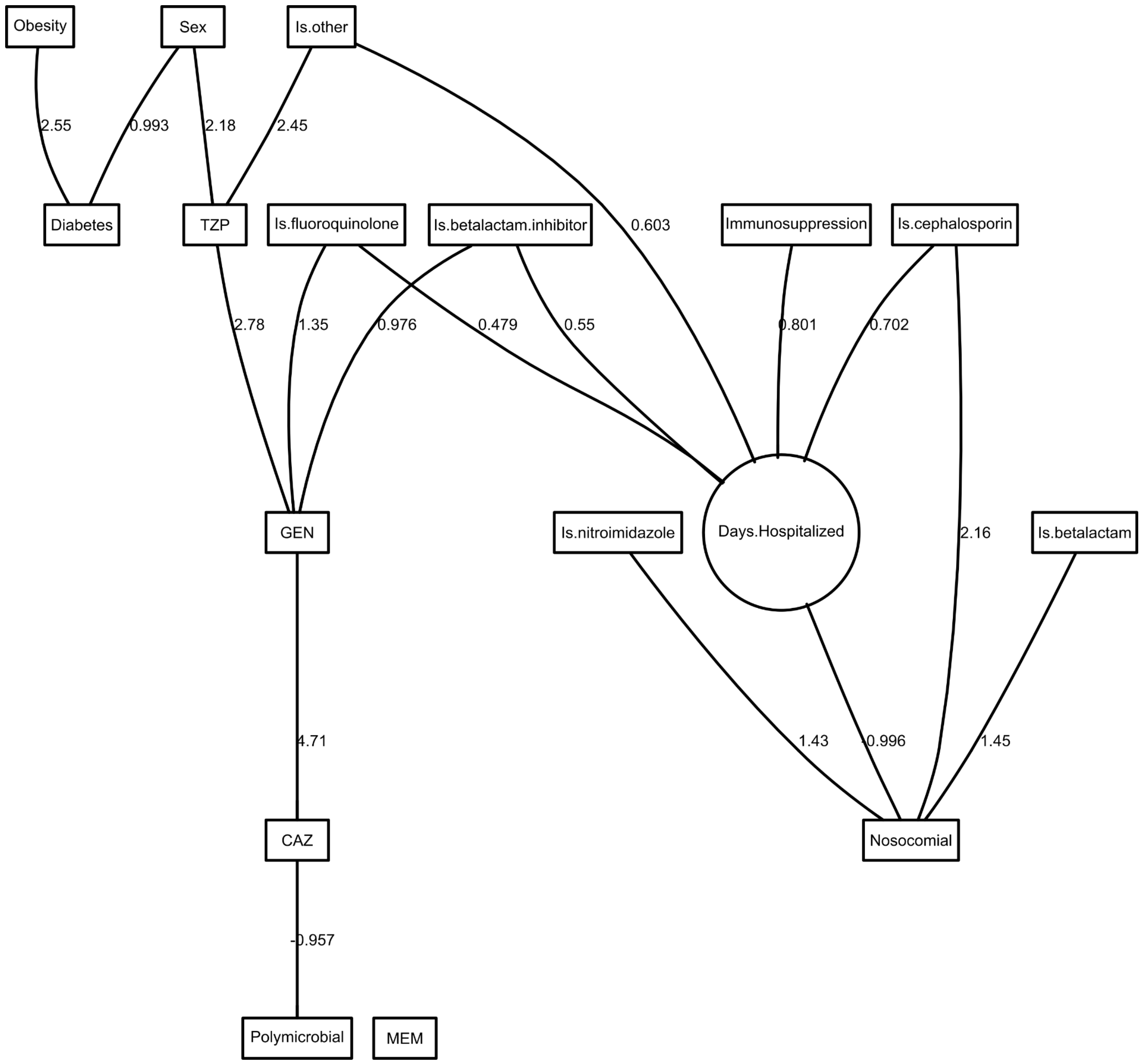

Figure S9: Final DAG for *P aeruginosa* in wound.

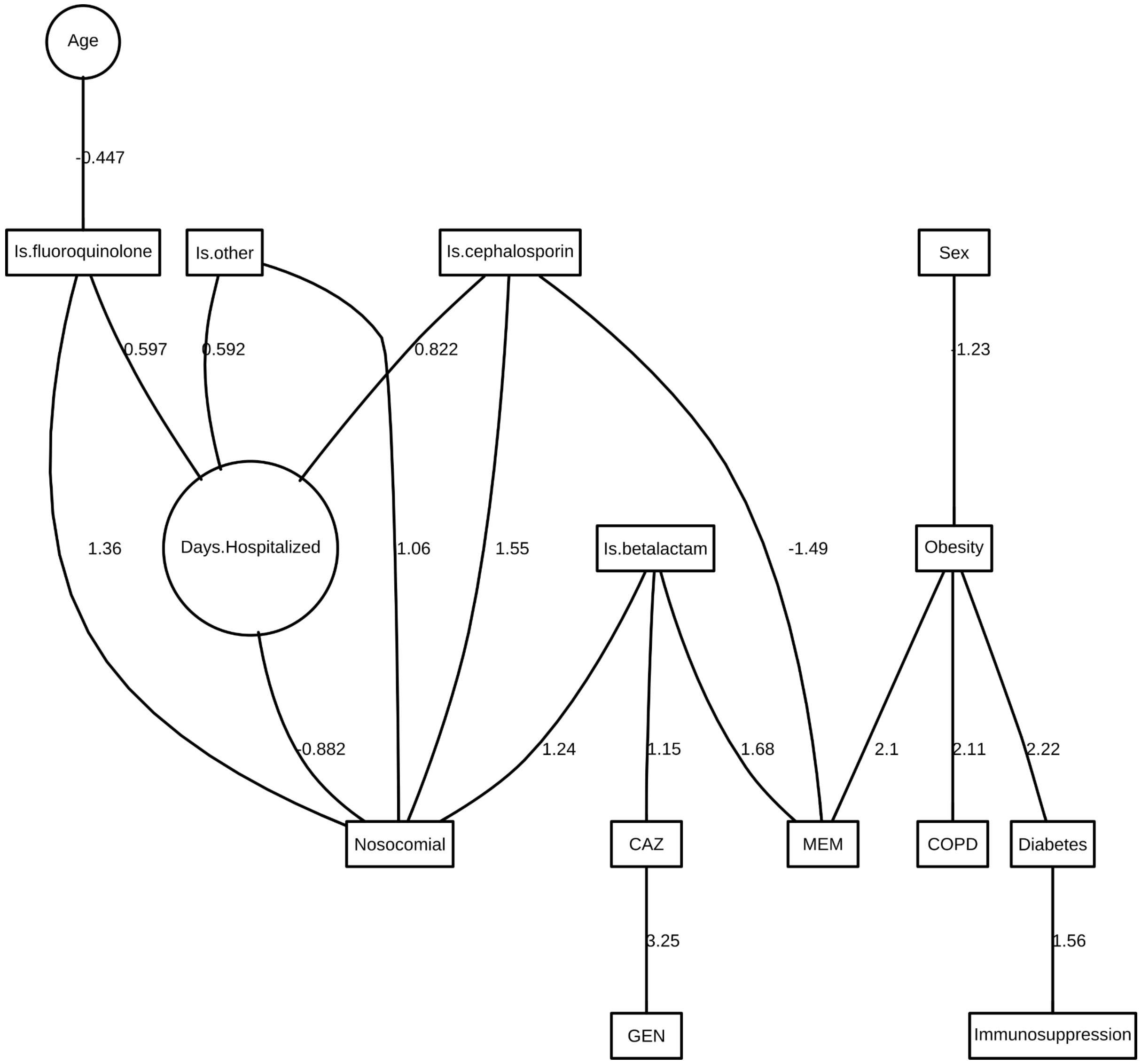

Figure S10: Final DAG for *P aeruginosa* in sputum.

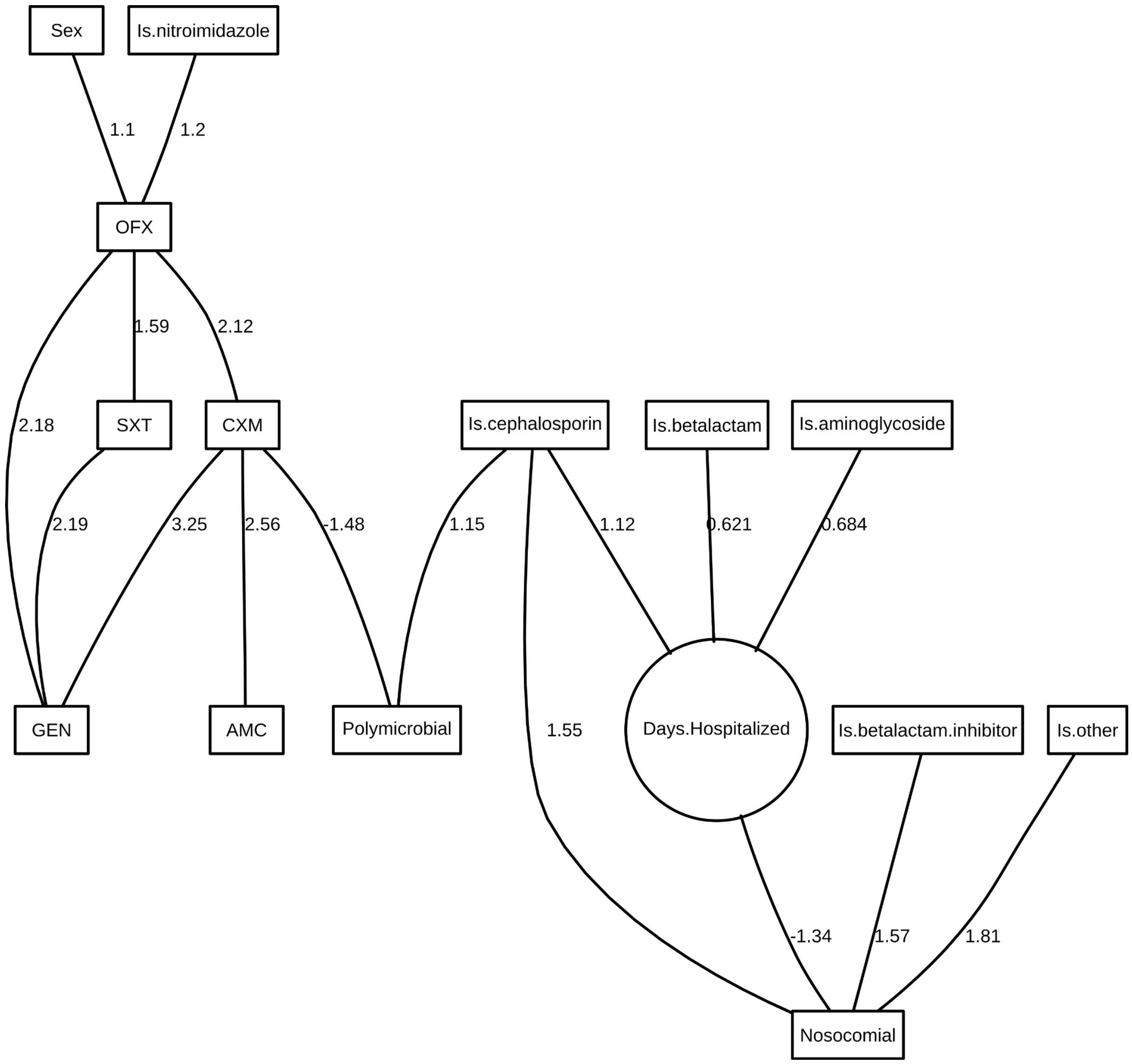

Figure S11: Final DAG for *P mirabilis* in urine.

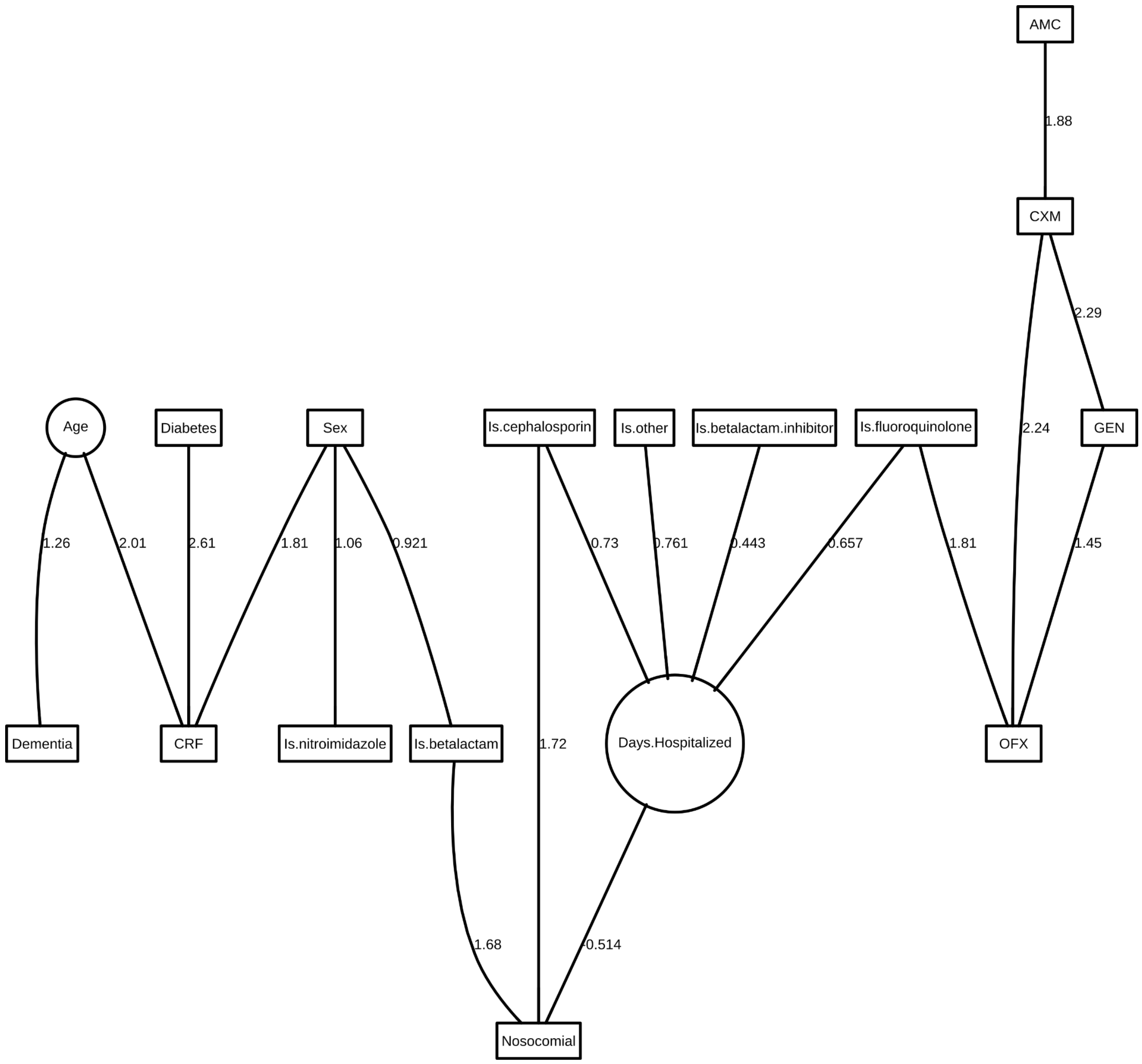

Figure S12: Final DAG for *P mirabilis* in wound.

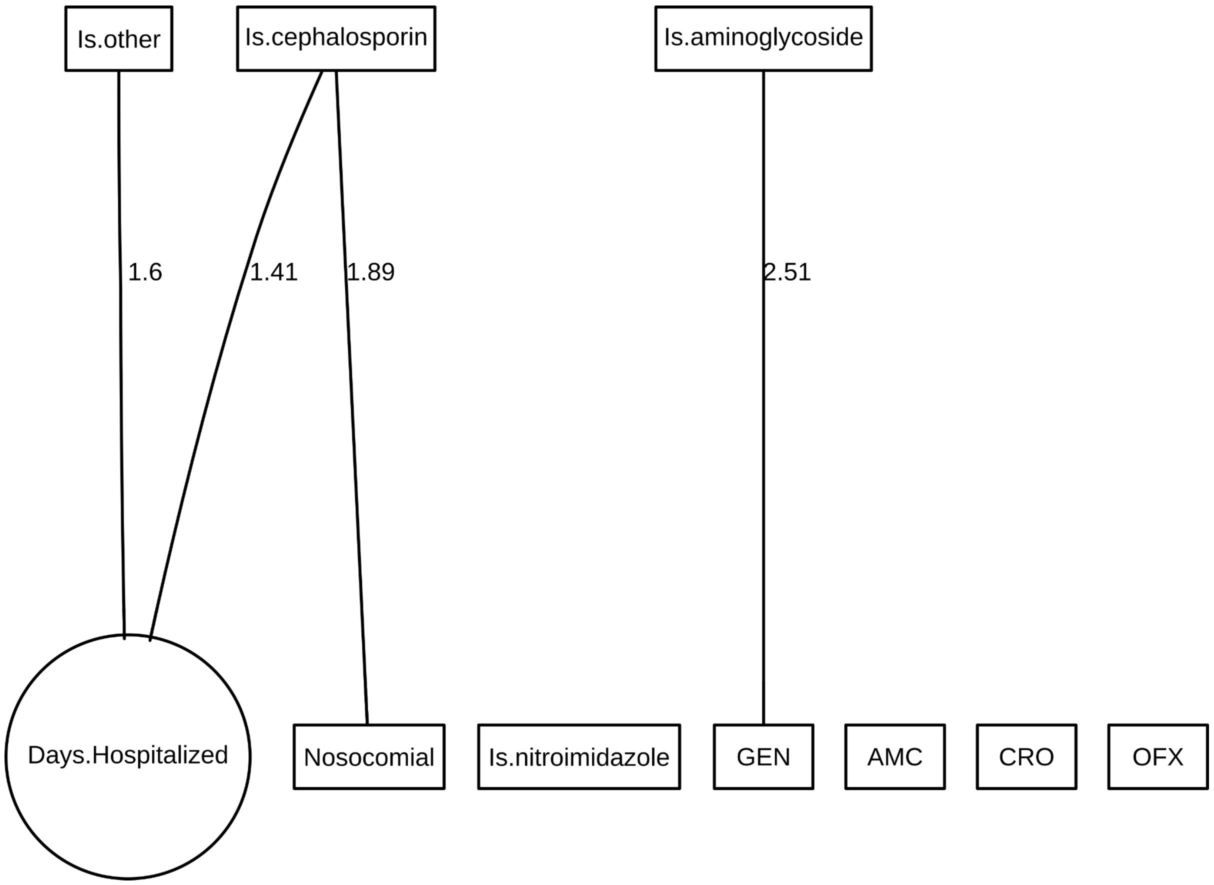

Figure S13: Final DAG for *P mirabilis* in aerobic blood.

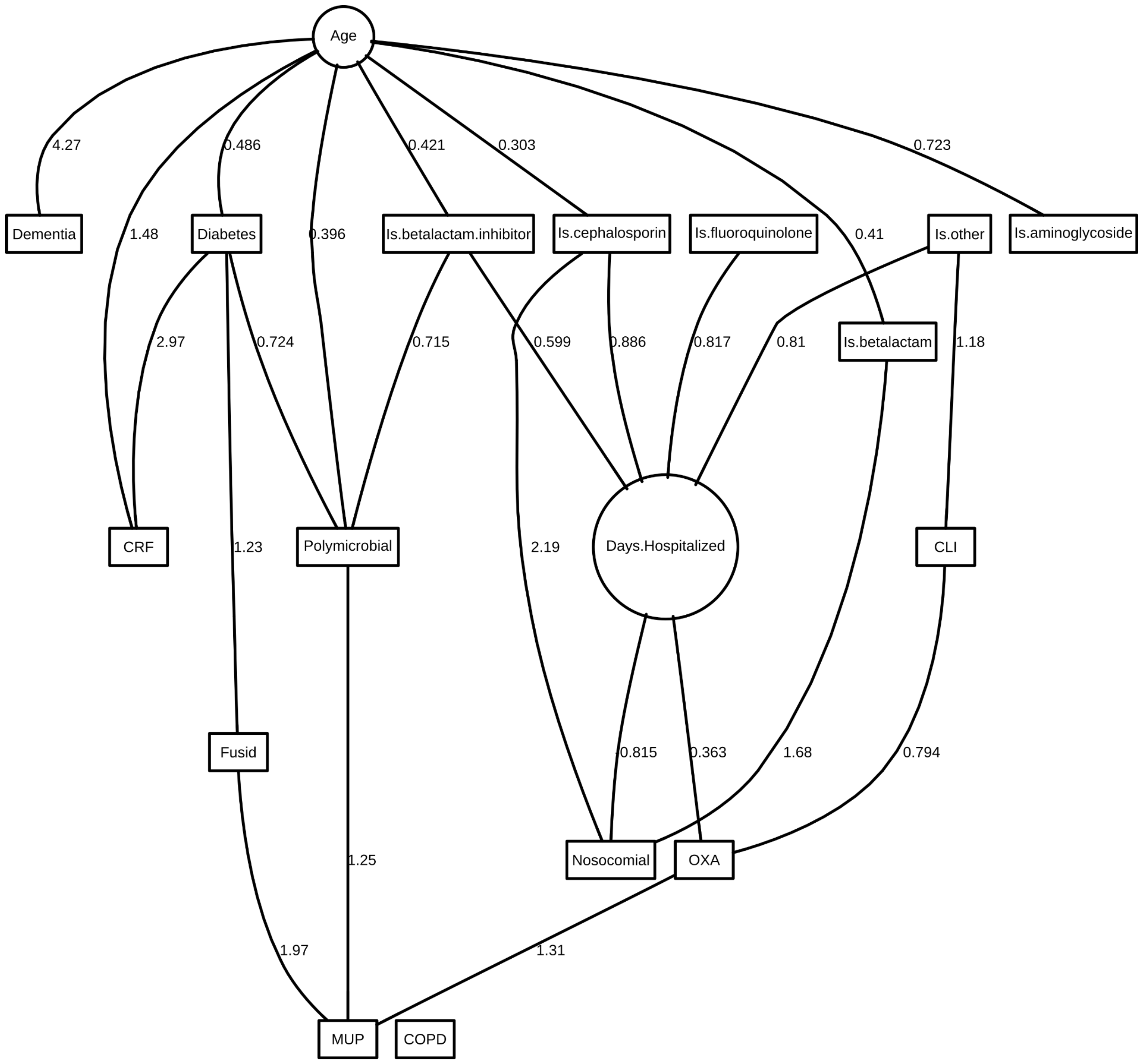

Figure S14: Final DAG for *S aureus* in wound.

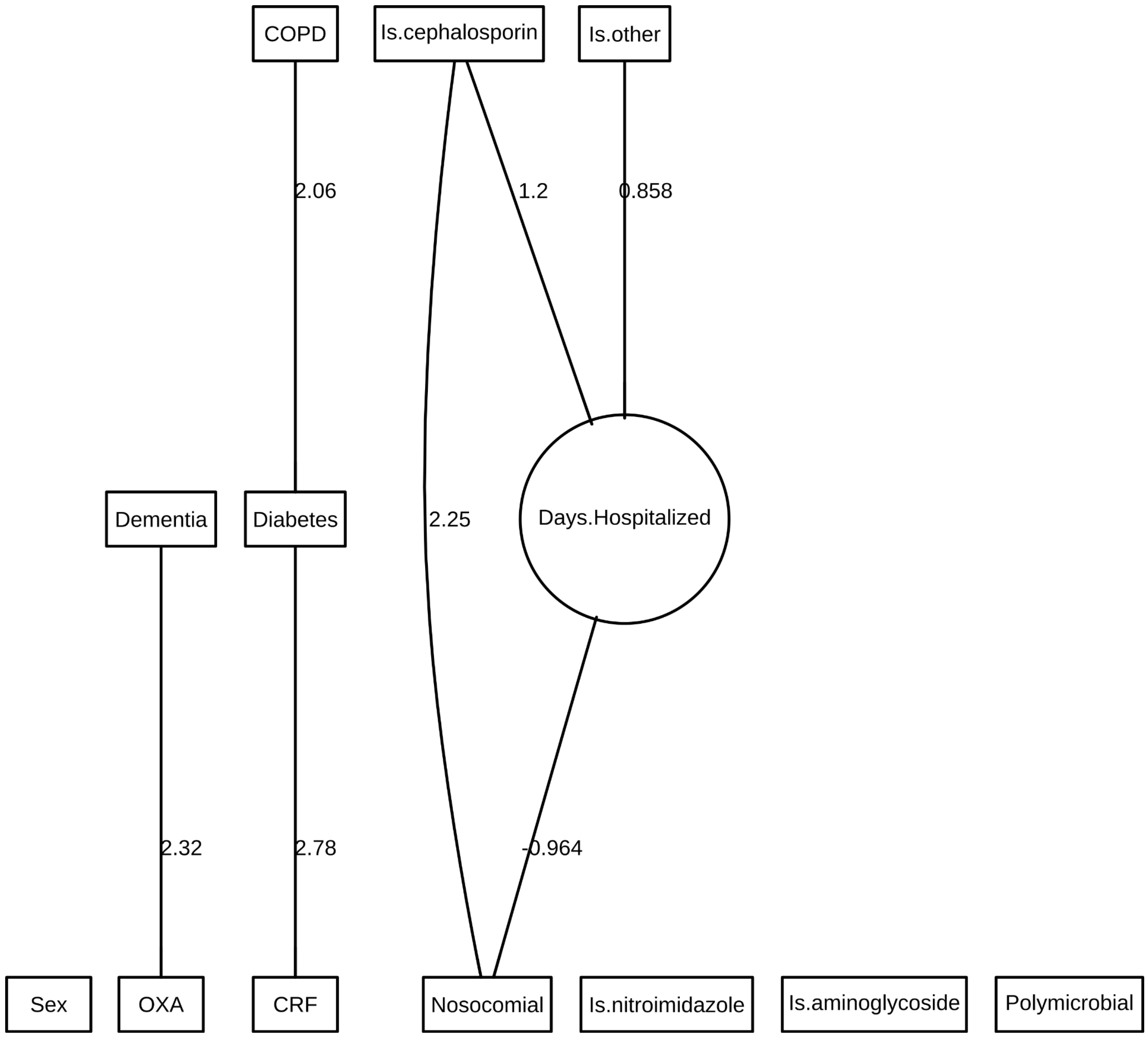

Figure S15: Final DAG for *S aureus* in aerobic blood.

Table S3: Median difference and confidence intervals between parameter estimates (ln(OR)) obtained from models of bacterial species cultured from different sources.

		Confidence interval of difference				
		2.50%	50%	97.50%		
E coli						
CAZ,GEN						
	Urine - Wound	-0.16	0.06	0.27	NS	
CAZ,OFX						
	Aerobic - Urine	-0.25	0.09	0.44	NS	
	Aerobic - Wound	0.87	1.00	1.15		
	Urine - Wound	0.71	0.92	1.12		
GEN,OFX						
	Aerobic - Urine	0.86	1.33	1.87		
	Aerobic - Wound	0.70	0.97	1.31		
	Urine - Wound	-0.56	-0.36	-0.15		
K pneumoniae						
GEN,OFX						
	Aerobic - Urine	0.95	2.05	3.66		
	Aerobic - Wound	0.58	1.24	2.34		
	Urine - Wound	-1.33	-0.81	-0.37		
OFX,TZP						
	Urine - Wound	-1.46	-0.78	-0.27		
P aeruginosa						
CAZ,GEN						
	Urine - Sputum	0.42	0.57	0.76		
	Wound - Sputum	1.48	1.50	1.68		
	Urine - Wound	-1.25	-0.93	-0.74		
P Mirabilis						
Urine - Wound						
	AMC,CXM	0.63	0.70	0.84		
	CXM,GEN	0.83	1.04	1.37		
	CXM,OFX	-0.26	-0.14	-0.01	NS*	
	GEN,OFX	0.64	0.78	1.06		

Note: AMC, amoxicillin/clavulanate; CAZ, ceftazidime; CXM, cefuroxime; GEN, gentamicin; OFX, ofloxacin; TZP, piperacillin/tazobactam. NS denotes no significant difference in parameter estimate between bacterial sources. *Effectively not significant.
